# Effects of food nutrition labels on the health awareness of school-age children

**DOI:** 10.1186/s12889-022-13613-y

**Published:** 2022-06-24

**Authors:** Ching-Yi Wang, Chung-Jia Hsu, Dengchuan Cai

**Affiliations:** 1 Department of Creative Product Design, Asia University, No. 500, Lioufeng Rd., Wufeng, Taichung City, 41354 Taiwan; 2 Department of Visual Communication Design, Asia University, No. 500, Lioufeng Rd., Wufeng, Taichung City, 41354 Taiwan; 3 Department of Industrial Design National, Yunlin University of Science and Technology, No.123, Sec. 3, University Rd, Douliou, Yunlin City, 64002 Taiwan

**Keywords:** Front of package (FOP) nutrition labeling, Guideline daily amounts (GDA), Traffic light system (TLS), Warning label, Health awareness

## Abstract

**Background:**

Overweight and obesity have been described as a global epidemic that seriously affects the health of adults and children. Front of Package (FOP) Nutrition Labeling can increase consumers’ awareness of unhealthy foods. The purpose of this study is to find effective deterrence and improve children’s health awareness via the FOP.

**Methods:**

This study examined children’s health awareness of snack packaging using the four labels: guideline daily amounts (GDA), traffic light system (TLS), Apple label (designed in this study), and Warning label. This study recruited 343 children in the sixth grade, including 223 children living in cities and 120 children living in rural areas. First, 30 children in grades 3 to 6 selected 8 snacks that they often buy. Then, each snack was synthesized into these four labels according to their nutritional content for a total of 32 samples. Finally, a questionnaire was used to evaluate the health of snack packaging and the visibility of nutrition labels.

**Results:**

Four results can be drawn: (1) GDA, Apple label and TLS can help children determine healthier snack choices, (2) black Warning label cannot induce children to make healthier choices, (3) children who often buy snacks have low health awareness, and (4) rural children have weak health awareness of snack packaging.

**Conclusions:**

These results can provide a packaging label design, which can effectively improve children’s health awareness.

**Supplementary Information:**

The online version contains supplementary material available at 10.1186/s12889-022-13613-y.

## Background

The overweight and obesity risks have been described as a global epidemic affecting adults and children in both developed and developing countries [1–5]. In particular, foods with high edible sugar, fat and salt have been considered as the most important food factors for promoting non-conductive diseases associated with weight gain, obesity and diet [6]. Front of Package (FOP) Nutrition Labeling can effectively encourage the food industry to re-develop their products and develop new and healthier foods [7–13]. However, most research on the effectiveness of the FOP focuses on adults [12, 14]. Nutrition labels have no significant effects on children’s choice of food [15, 16]. Most children’s foods contain high sugar, sodium and fat content, and these foods are typically sold through cartoon characters in ads and their packaging [17, 18]. Children’s products usually promote entertainment and health with bright colors, cartoon characters, cute patterns, nutrition promotion, natural food images (such as fruit pictures), and descriptions of physical activities (implying the power or intensity of product consumption) [19–28]. Children are extremely susceptible to these marketing strategies. In addition, food packaging (such as name, shape, color, flavor, and characters) generally regarded by children as “interesting” is more praised than the taste of “uninteresting” food [28, 29]. In other words, when children’s favorite cartoon patterns are printed on the packaging, or the color of the packaging attracts their attention, these may cause children to disregard the health results of the food. In addition, there is evidence that urban-rural gap factors can also affect children’s health [30–35]. Increasing the educational level of women in rural areas may have a greater impact on reducing child malnutrition [34, 36, 37]. Therefore, family environment and education seem to potentially affect children’s health awareness.

Front of Package (FOP) Nutrition Labeling improves consumers’ ability to correctly identify healthy products and encourage people to choose more healthy foods [38–43]. However, children may not be familiar with FOP. Many studies have investigated the effectiveness of the FOP [44–48]; there is evidence that the possible explanation for the lack of health perceptions of most children with FOP is that their evaluations are based on previous preconceived perceptions of product and label design, rather than relying on nutritional information [34, 49] point out that familiarity with the labeling system may affect consumers’ willingness to use these products. In short, consumers will not choose unfamiliar nutrition labels; this is especially important for children because they rarely use nutritional information to guide their food choices [50]. The most common forms of FOP include guideline daily amounts (GDA), traffic light system (TLS), and Warning labels. GDA uses symbols to display the number of specific nutrients or calories per serving, providing a semi-directive assessment of nutritional quality. Based on GDA, TLS adds traffic light colors (red, yellow, and green) to indicate the level of nutrients in food [12]. A significant amount of evidence shows that TLS seems to be more acceptable than GDA to consumers; this may be due to the color form enabling consumers to identify and use the information easily and more quickly [51]. These colorful labels can help avoid the consumption of unhealthy foods [52, 53]. In addition, black Warning labels [34, 40, 54, 55] indicate that the content of key nutrients (such as salt, sugar, and saturated fat) is higher than the standard, which can warn consumers that these foods contain a large amount of ingredients that cause obesity, thereby helping to prevent consumers from buying unhealthy products [40, 56, 57]. On the contrary, the way TLS classifies low/medium/high nutrient content conveys contradictory information, that is, the product may contain a high content of one nutrient and low content of other nutrients [58]. Food packaging with low nutrient content information (the label is green) can easily mislead people into thinking that these products are healthy [59–64].

There are still many controversies about the promotional effects of the nutritional content noted on these food labels. This study questions whether these labels are effective in deterring children from buying unhealthy foods. In addition, children are easily tempted by food marketing as attractive label patterns (such as fruits or cartoons) may easily attract children’s attention. Therefore, this study compares the health perception and impact of snack packaging containing four FOP forms on children: GDA, Apple label (designed in this study), TLS, and Warning label. These results may provide an FOP improvement plan and a new marketing strategy for children’s food.

## Methods

### Participants

Three hundred forty-three sixth-grade Taiwanese children (158 males, 185 females, M = 11.49 years old, SD = 0.50 years old) (Table 1) were recruited to conduct health assessments based on different packaging labels. Among them, 223 children live in cities (108 males and 115 females, M = 11.48 years old, SD = 0.50 years old) and come from Tainan City’s Dawan Elementary School. In addition, 120 children live in rural areas (50 males and 70 females, M = 11.52 years old, SD = 0.50 years old) and come from Puli Elementary School in Nantou County.

**Table 1:** Participant information, including: gender, age, and frequency of snack consumption in urban and rural areas

Item	Urban	Rural	All
Gender			
Male	108	50	158
Female	115	70	185
Age	11.48	11.52	11.49
Frequency of snack consumption			
Type 1 (never ~ once a month)	83	35	118
Type 2 (more than twice a month ~ once a week)	101	43	144
Type 3 (more than twice a week ~ every day)	39	42	81

### Material

#### Label settings

**Fig. 1:**
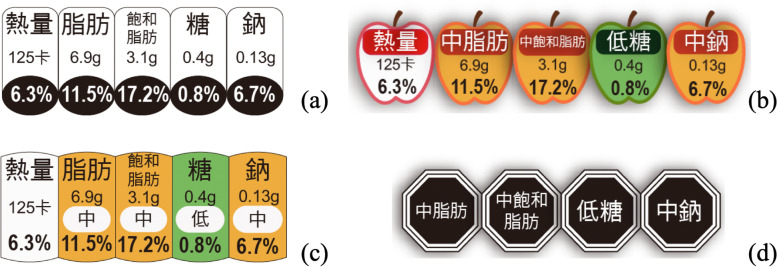
Nutrition labels on the front of the package: **a** GDA: Colored in black and white and showing the number of specific nutrients or calories per serving, **b** Apple label (designed in this study): Combining GDA and TLS features plus apple-patterned outlines; **c** TLS: Based on GDA, TLS adds traffic light colors (red, yellow, and green) to indicate nutrient levels, and **d** Warning label: Black octagon with nutrients showing exceeding standard contents

Figure 1 shows four kinds of nutrition labels: GDA, Apple label, TLS, and Warning label. All the labels were located on the left or right under the front of the package. The GDA, Apple label, and TLS display the content of each serving and the percentage of the recommended daily intake, while the Warning labels only display warning text for low, medium, and high nutrition. The Apple label designed by this study is based on the appearance of the TLS label changed to a fruit pattern.

#### Packages

Thirty children in grades 3 to 6 (9 males, 21 females, M = 11.47 years old, SD = 1.01 years old) chose eight kinds of snacks that are most popular with Taiwanese children (see the Additional file 1): Kola Nuts, Guaiguai (5 flavors), Fruit Jelly (Guaiguai soft candies), Lay’s Potato Chips (original flavor), Scientific Noodles, Jinsha Chocolate, Cheetos (cheese), and Pocky (chocolate).

**Fig. 2:**
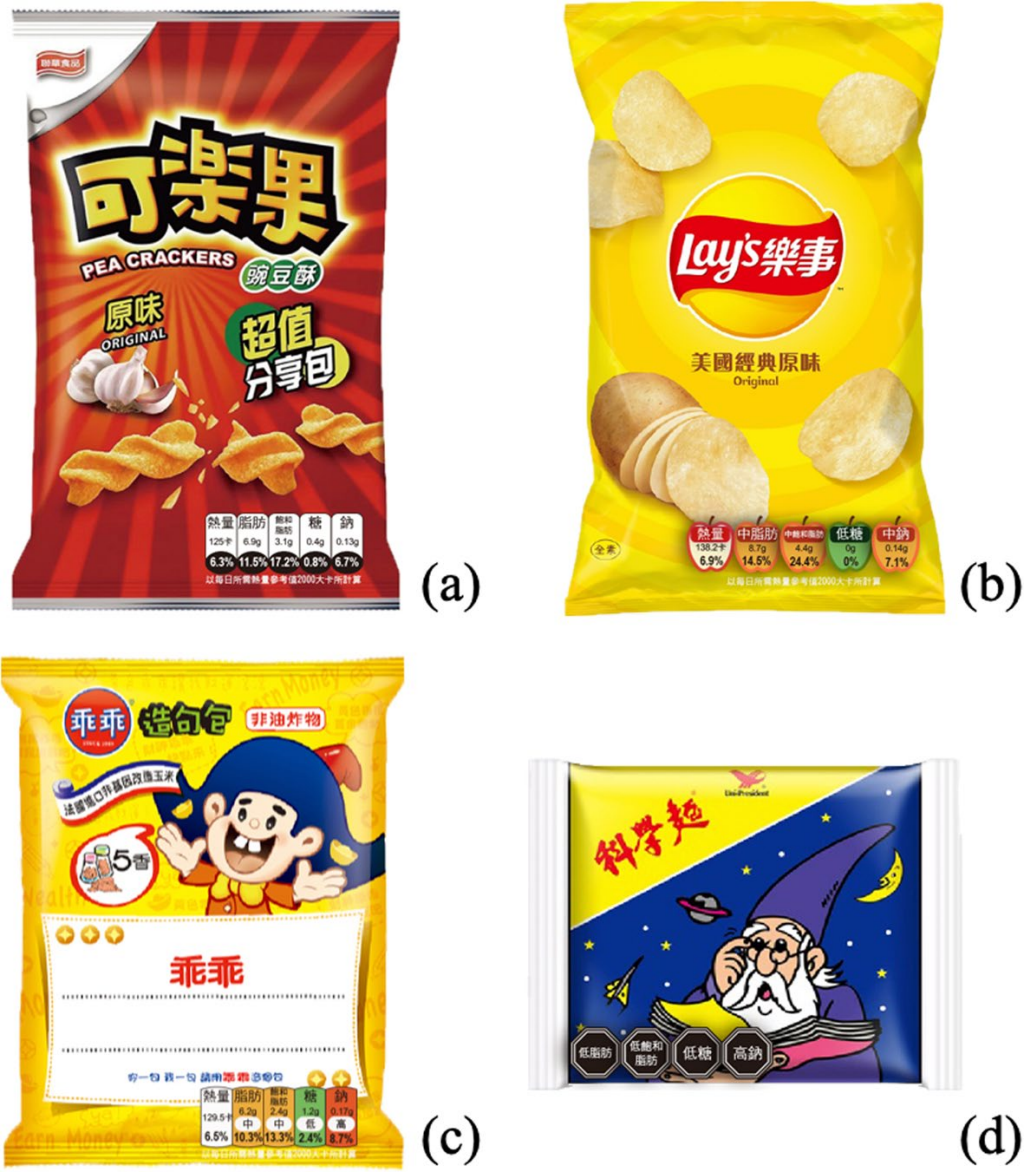
Snack packaging contains these four examples of labels on the bottom left or right: **a** “Kola Nut” with GDA label; **b** “Lay’s Potato Chips” with Apple label; **c** “Kuai Kuai” with TLS label, and **d** “Science Noodles” with Warning label

Figure 2 shows examples of the positions and sizes of the four labels displayed on the packaging. Each package uses a commercially available original image, and synthesizes four nutrition labels on the front package. All the labels ware located at the bottom of the front packaging, and their exact location (left or right) depends on the original packaging design. All the packaging images were presented in the same size.

### Questionnaire

The content of the questionnaire was divided into three parts: (1) personal information, (2) packaging health evaluation, and (3) label visibility evaluation. The evaluation scale uses a 7-point Likert scale (1 = completely unhealthy, 7 = completely healthy). In order to avoid the psychological burden of children caused by the large number of pictures, these samples were evaluated by 5 groups (see Table 2). Each group judged 8 pictures. Groups 1 to 4 evaluated the images containing four labels (GDA, Apple label, TLS, and Warning label) and the visibility of the four labels. The last group evaluated the original packaging without any label, and did not need to denote the visibility of the label. Samples between groups were not repeated.

**Table 2:** The content of the questionnaire includes personal information and evaluations of packaging healthiness and label visibility. The sample types and order of appearance were divided into five groups for healthiness and visibility assessment

Item	Content	
(1) Personal information	Gender, Age, and Consumption Frequency in Snacks	
(1)(1)(1)(1)(2) Packaging health evaluation (package + label)	Group 1	A+(a), B+(b), C+(c), D+(d), E+(a), F+(b), G+(c), and H+(d)
	Group 2	A+ (b), B+(c), C+(d), D+(a), E+(b), F+(c), G+(d), and H+(a)
	Group 3	A+(c), B+(d), C+(a), D+(b), E+(c), F+(d), G+(a), and H+(b)
	Group 4	A+(d), B+(a), C+(b), D+(c), E+(d), F+(a), G+(b), and H+(d)
	Group 5	A, B, C, D, E, F, G, and H without label
(3) Label visibility evaluation	Group 1–4	(a), (b), (c), and (d) labels

Packaging code: A (Kola Nut), B (Kuai Kuai), C (Kuai Kuai: QQ Fruit Jelly), D (Lay’s Potato Chips: Original), E (Science Noodles), F (Ferrero Rocher Chocolate), F (Cheetos: Cheese), and H (Pocky: Chocolate)

Nutrition label code: (a) GDA, (b) Apple label, (c) TLS, and (d) Warning label

### Procedure

Before the experiment, children were asked to sit in front of a 19-in. screen. The researchers explained the contents of the experiment to the children. At the beginning of the experiment, each package picture was displayed in the center of the screen with a white background. The sample has 40 pictures in total. During the experiment, no information about packaging were provided to the children. All the children used computers to complete tasks under the supervision of the researcher. If children had questions, the researcher helped them.

### Data analysis

First, the data was analyzed using univariate analysis. The “healthiness” of the package and the “visibility” of the label were dependent variables. The “nutrition label” (GDA, Apple label, TLS, Warning label, and No label (standard)), the “consumption frequency” of snacks (Type 1, Type 2, and Type 3), and the “residential area” (urban and rural) were the independent variables. If the ANOVA results significantly differed, post hoc tests were analyzed by Scheffe. Then, a paired sample T-test was used to compare potentially significant factors.

Finally, linear regression analysis was performed to predict children’s health awareness of the five labels on the packaging. The “consumption frequency”, “residential area”, and “gender” were set as dependent variables. The “healthiness” and “visibility” were the independent variables. The scatter plot examined the linear relationship between the response variables and the standardized residuals.

## Results

### ANOVA results

#### Health evaluation of snack packaging

Table 3 shows the overall ANOVA results; different nutrition labels, consumption frequency, and residential area affect children’s health awareness of snack packaging (F[4, 1586] = 4.77, *p* = 0.001; F[2, 1586] = 22.95, *p* = 0.000; F[1, 1586] = 47.34, *p* = 0.000, respectively). The following further examines the health awareness of these three factors on packaging through post hoc tests and T-test comparisons.

**Table 3:** The overall ANOVA results of different health awareness of packaging among the nutrition labels, consumption frequency, and residential area

Factor	DF	MS	F	Sig.
Nutrition label	4	6.64	4.77	0.001**
Consumption frequency	2	31.97	22.95	0.000**
Residential area	1	65.95	47.34	0.000**
Nutrition label x Consumption frequency	8	2.06	1.48	0.161
Nutrition label x Residential area	4	1.87	1.35	0.251

^*^p < 0.05

^**^p < 0.001

##### Differences between the nutrition labels

**Fig. 3:**
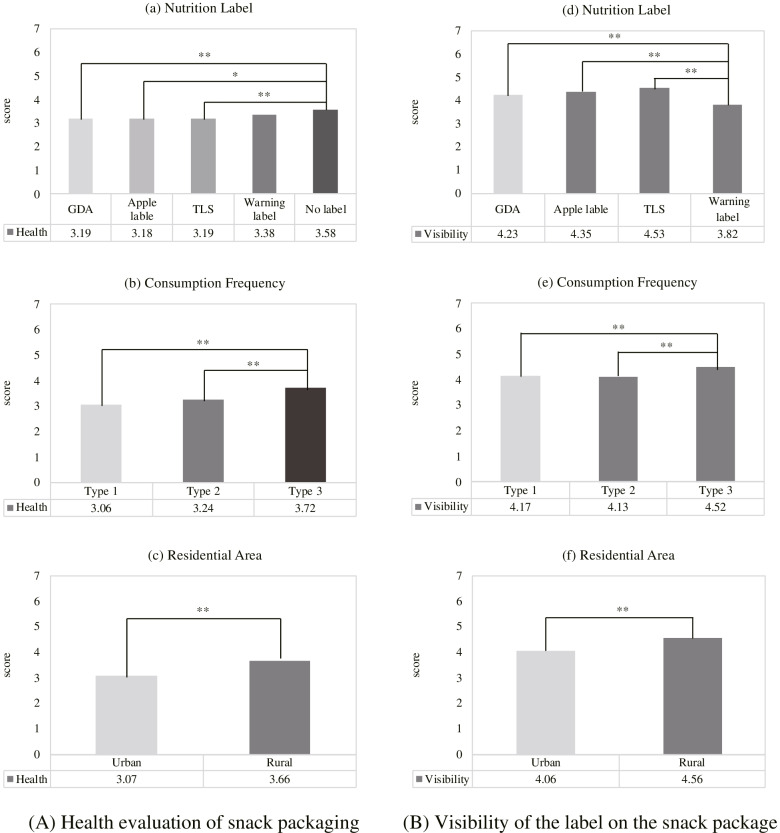
Mean and T-test evaluation results of children’s health awareness of packaging and visibility of labels on the nutrition labels, consumption frequency, and residential area

The results of the post hoc test showed that GDA, Apple label, TLS and Warning labels can be grouped into one group, while Warning label and no label comprise another group. The comparison results of the T test (Fig. 3a) found that no label (score = 3.58), compared with GDA (score = 3.19), Apple label (score = 3.18) and TLS (score = 3.19) has significant differences (*p* = 0.003, *p* = 0.003, and *p* = 0.004, respectively). However, there is no significant difference between Warning label (score = 3.38) and no label packaging (*p* = 0.409). Because the main function of FOP is to prevent unhealthy snacks, the Warning label (GDA and TLS) has a lower score than the no label, indicating that these snacks have been successfully promoted as unhealthy. The health prevention of Warning label is worse than the other three labels, and has almost the same effect as no label.

##### Differences of consumption frequency

According to the results of post hoc tests, the consumption Type 1 and Type 2 belong to one group, while the consumption Type 3 is another group. The comparison results of the T test (Fig. 3b) showed that children who often buy snacks (score = 3.72) have lower health awareness (all *p* < 0.000), compared to children who belong to consumption Type 1 and Type 2 (score = 3.06 and score = 3.24, respectively). This means that children who often buy snacks are less aware of the health implications of snacks, and other children agreed that these are unhealthy snacks.

##### Differences of residential area

Children living in urban areas (score = 3.07) have lower health ratings of snack packaging compared to rural children (score = 3.66) (*p* = 0.000) (Fig. 3c), indicating that urban children have a stronger health awareness of snack packaging.

#### Visibility of the label on the snack package

Table 4 shows the overall ANOVA results found that different nutrition labels, consumption frequency, and residential area affect the visibility of children’s snack packaging (F[31348] = 9.87, *p* = 0.001; F[21348] = 3.26, *p* = 0.039; F[11348] = 21.25, *p* = 0.000, respectively). The following further examines the visibility of these three factors on packaging through post hoc tests and T-test comparisons.

**Table 4:** The overall ANOVA results of different visibility of the label among the nutrition label, consumption frequency, and residential area

Factor	DF	MS	F	Sig.
Nutrition label	3	25.42	9.87	0.000**
Consumption frequency	2	8.40	3.26	0.039*
Residential area	1	54.75	21.25	0.000**
Nutrition label x Consumption frequency	6	1.82	0.71	0.65
Nutrition label x Residential area	3	0.30	0.12	0.95

^*^p < 0.05

^**^p < 0.001

##### Differences between the nutrition labels

The results of the post hoc test revealed that the GDA, Apple, and TLS tags are classified into one group, and the Warning label is another group. The comparison results of the T test (Fig. 3d) showed that the Warning label (score = 3.82) significantly differs from the GDA (score = 4.23), Apple label (score = 4.35) and TLS (score = 4.53) (*p* = 0.01; *p* = 0.000 and *p* = 0.000, respectively).

##### Differences in consumption frequency

According to the results of post hoc tests, the consumption Type 1 and Type 2 belong to one group, while the consumption Type 3 is another group. The comparison results of the T test (Fig. 3e) revealed that children who meet the consumption Type 3 (score = 3.52) consider that nutrition labels have a higher degree of visibility (all *p* < 0.000), compared to children who belong to consumption Type 1 and Type 2 (score = 3.17 and score = 3.13, respectively). This means that children who often buy snacks do notice the existence of nutrition labels, albeit they still buy the snacks. It may be that they have significantly low health awareness.

##### Differences of residential area

Children living in urban areas (score = 4.06) are less likely to pay attention to nutrition labels on packaging i.e., these appear less visible compared to children in rural areas (score = 4.56) (*p* < 0.000) (Fig. 3f).

### Regression results

**Fig. 4:**
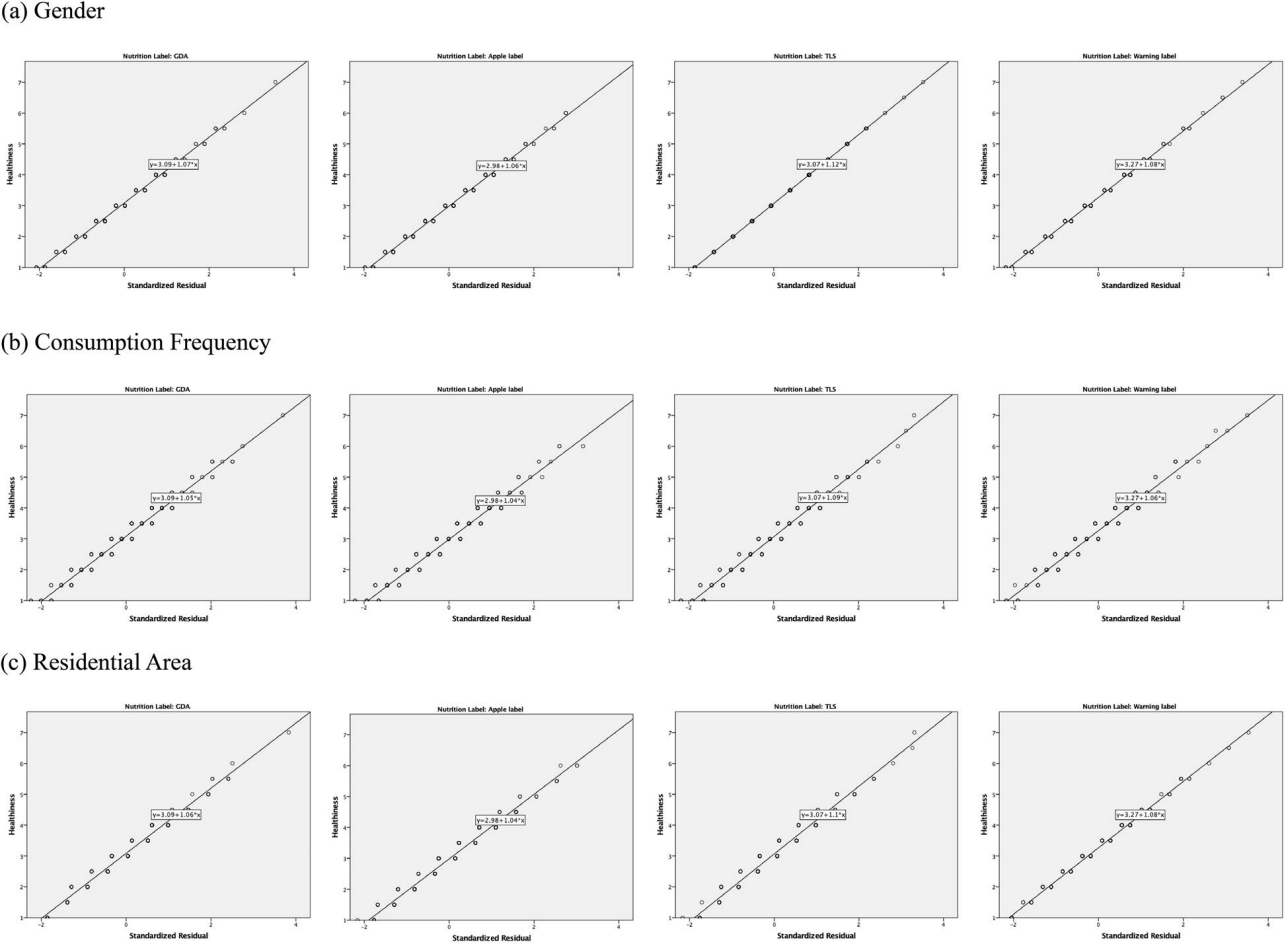
The scatter plots of “healthiness” for the four labels, consumption frequency, and residential area between healthiness and the standardized residuals

**Fig. 5:**
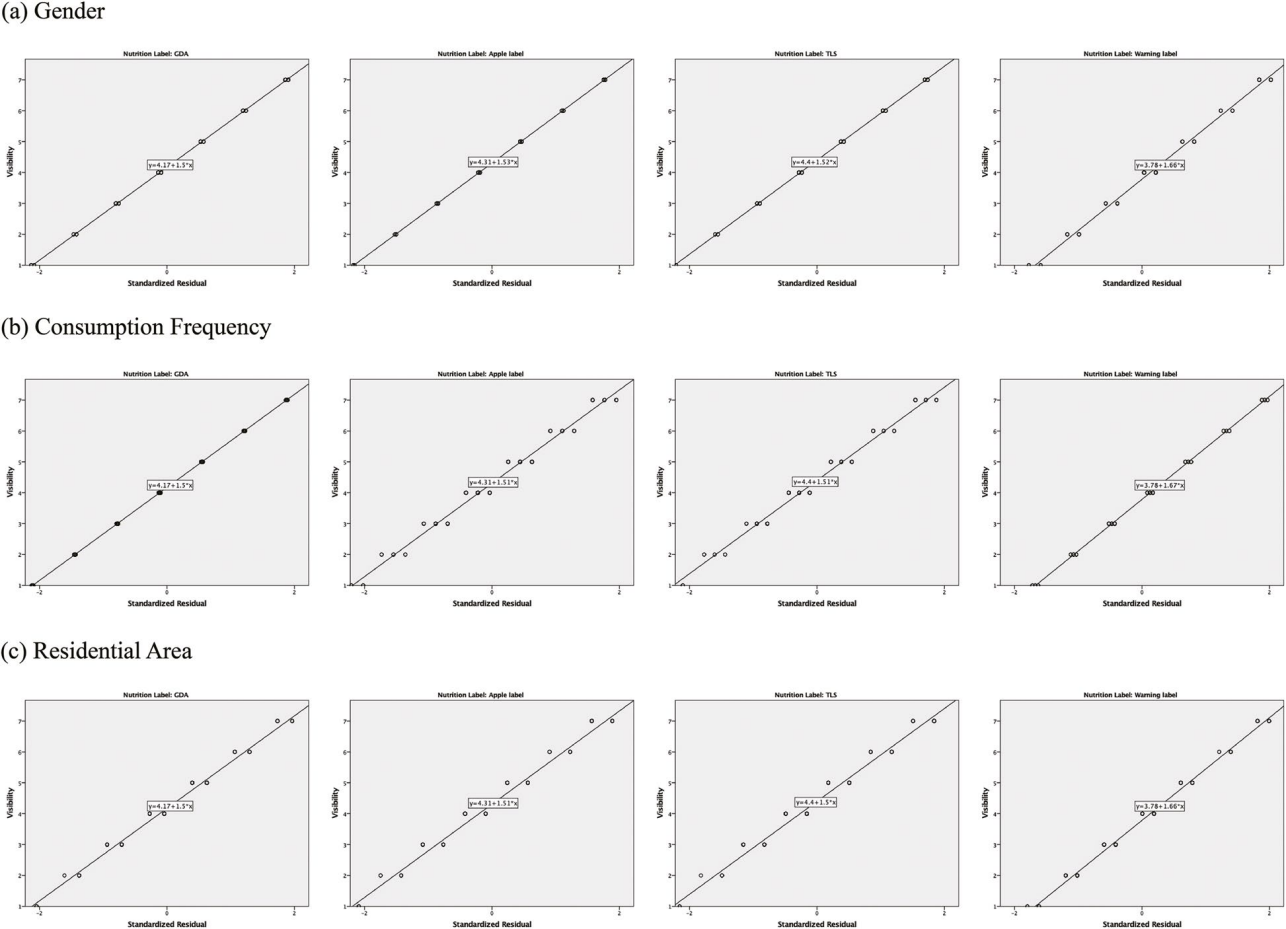
The scatter plots of “visibility” for the four labels, consumption frequency, and residential area between healthiness and the standardized residuals

Tables 5 and 6 display the results of the “healthiness” and “visibility” coefficients for GDA, Apple Label, TLS, and Warning label in the gender, consumption frequency, and residential area variables. Figures 4 and 5 show the scatter plots of these four labels, consumption frequency, and residential area between visibility and the standardized residuals, respectively. These scatter plots illustrate the linear relationships between the response variables and the standardized residuals.

**Table 5:** The regression results of “healthiness” coefficients for GDA, Apple label, TLS, and Warning label in the gender, consumption frequency, and residential area variables

Factor	B	Beta	t	Sig.
GDA				
(Constant)	3.43		16.73	0
Gender	−0.22	−0.10	−1.74	0.083
(Constant)	2.61		15.46	0
Consumption Frequency	0.25	0.18	3.06	0.002*
(Constant)	2.56		13.60	0
Residential Area	0.40	0.17	2.97	0.003*
Apple label				
(Constant)	3.29		16.22	0
Gender	−0.20	−0.10	−1.62	0.107
(Constant)	2.43		14.63	0
Consumption Frequency	0.29	0.21	3.57	0.000**
(Constant)	2.45		13.12	0
Residential Area	0.41	0.18	3.06	0.002*
TLS				
(Constant)	3.09		14.41	0
Gender	−0.01	−0.01	−0.10	0.923
(Constant)	2.52		14.39	0
Consumption Frequency	0.29	0.20	3.42	0.001**
(Constant)	2.48		12.67	0
Residential Area	0.45	0.19	3.22	0.001**
Warning label				
(Constant)	3.51		16.94	0
Gender	−0.16	−0.07	−1.22	0.224
(Constant)	2.72		16.07	0
Consumption Frequency	0.29	0.20	3.48	0.001**
(Constant)	2.99		15.58	0
Residential Area	0.21	0.09	1.53	0.128

^*^p < 0.05

^**^p < 0.001

**Table 6:** The regression results of “visibility” coefficients for GDA, Apple label, TLS, and Warning label in the gender, consumption frequency, and residential area variables

Factor	B	Beta	t	Sig.
GDA				
(Constant)	4.07		14.07	0
Gender	0.07	0.02	0.38	0.701
(Constant)	4.21		17.49	0
Consumption Frequency	−0.02	−0.01	−0.17	0.863
(Constant)	3.72		13.93	0
Residential Area	0.34	0.11	1.81	0.072
Apple label				
(Constant)	4.25		14.50	0
Gender	0.04	0.01	0.20	0.840
(Constant)	3.77		15.60	0
Consumption Frequency	0.28	0.14	2.39	0.017*
(Constant)	3.67		13.62	0
Residential Area	0.49	0.15	2.53	0.012*
TLS				
(Constant)	4.30		14.71	0
Gender	0.07	0.02	0.37	0.712
(Constant)	3.93		16.27	0
Consumption Frequency	0.25	0.13	2.11	0.036*
(Constant)	3.74		13.96	0
Residential Area	0.50	0.15	2.60	0.010*
Warning label				
(Constant)	4.25		13.32	0
Gender	−0.31	−0.09	−1.54	0.125
(Constant)	3.64		13.66	0
Consumption Frequency	0.07	0.03	0.57	0.569
(Constant)	3.39		11.42	0
Residential Area	0.30	0.09	1.43	0.155

^*^p < 0.05

^**^p < 0.001

#### Prediction of healthiness

All of the labels (*p* = 0.002, *p* = 0.000, *p* = 0.001, and *p* = 0.001, respectively) have strongly significant differences in consumption frequency. GDA, Apple Label, and TLS (*p* = 0.003, *p* = 0.002, and *p* = 0.001, respectively) showed significant differences in the residential area, but not the Warning label (*p* = 0.128). Nevertheless, there is no significant difference in gender. These regression results show that the consumption frequency is a better predictor of the healthiness of snack packaging than other factors, regardless of the nutrition label used in the packaging design. However, the gender factor did not help to predict the children’s health awareness for snack packaging.

#### Prediction of visibility

Both the Apple label and TLS exhibit significant differences in the consumption frequency and residential area (Apple label: *p* = 0.017 and *p* = 0.012; TLS: *p* = 0.036 and *p* = 0.010). However, GDA and Warning label do not have any significant factor. Also, there was no significant difference in gender. These results suggest that the higher color rendering of the Apple label and TLS makes them more predictable for package visibility compared to the other two labels.

## Discussion

### GDA, apple label, and TLS can effectively improve children’s health awareness

Front of Package (FOP) Nutrition Labeling was developed to enable consumers to choose healthy foods. This study proves that snack packaging with FOP is better than non-labeled snack packaging, as it can effectively improve children’s health awareness. This study unanimously agrees that past studies believe that indicating FOP on snack packaging can indeed change consumers’ health perception and their food choices [37, 39–43]. Because this visual cue highlights compliance with specific nutritional standards, it lets children know whether these products are healthy or unhealthy. This study cannot completely agree with previous studies that most children lack health awareness of FOP [34]. A possible explanation is that these sixth-grade participants are older. Children in this period might engage in logical thinking based on specific examples and have the ability to deal with consumption information [65]. Therefore, they could indeed perceive and process the nutritional information and health implications of these labels.

GDA, TLS and Apple label have the same effect of conveying nutritional information. Among them, the visibility of GDA is slightly lower than that of Apple label and TLS because GDA uses black and white labels. In contrast, the visual cue is not higher than the use of multi-color TLS and Apple labels. Packaging focuses on visual quality [28]. In the eyes of children, colorless GDA is regarded as “not interesting.” Because interesting packaging is usually better in regard to evaluation [28, 29]. In addition, the color information of TLS can be clearly conveyed to children. Because red can link warning and danger meanings to attract children’s attention, and effectively and quickly raise children’s health awareness [52, 53, 66–68].

Furthermore, snack packaging labels use fruit patterns to increase children’s attention and effectively convey the message that these products are unhealthy [19–21, 24]. Moreover, the health alertness of the Apple label can be effectively the same as that of GDA and TLS because the advantages of the Apple label’s pattern combine the color distinction of TLS and daily nutritional information of GDA. Graphic design is used by many businesses; they put cartoon or game characters on the packaging when selling snacks to increase children’s preference and brand attraction, leading children to buy unhealthy and high-calorie products [20]. Indeed, children are susceptible to these marketing strategies; therefore, the label design of traffic light colors and attractive shapes can effectively improve children’s health awareness.

### The controversy that warning label has no effective health warning

The Warning label did not achieve effective prompting in this study, and its effect is equivalent to “no label.” It is further found that the Warning label is less visible than the other three labels; this result is contrary to previous studies, namely that the Warning label can effectively deter consumers from choosing unhealthy products [34; 40). The display of the black Warning label is completely opposite to that of the other three nutrition labels. The information presented inside the Warning label is simple and only highlights unhealthy components [57].

This study speculates on three reasons. The first reason is the black of high color rendering of the Warning label, which only reveals the high content of information, but does not display other data. There is still insufficient information to provide more detailed information about “health”, which prevents children from being aware of the meaning of the warning. The second reason is the fact that children have regarded snacks as unhealthy, making snacks have no effect on Warning labels, a view which is consistent with [69], who found that soda water is the only category that has no warning effect. Similar studies, such as [57] also found that Warning labels only affect half of the participants, and did not produce any relevant effects on the remaining participants because consumers may often ignore the information on the label of unhealthy products [70]. The last reason may be based on different cultural perceptions. Since Taiwanese snack labels rarely exhibit the Warning label design, many children do not recognize this label. In Chile, the Warning label has been implemented in people’s basic food [34, 54, 55]. As this label is familiar and recognized by local people, Warning labels may not be suitable for Taiwanese snacks.

### Children who often buy snacks have low health awareness

Children who often buy snacks have significantly lower health awareness. This study speculates that the reason may be due to insufficient health education in the family or incorrect parenting methods, which leaves children with limited health knowledge [34, 36, 37]. In general, consumers’ food choices are usually affected by product health considerations [71]. When consumers are able to obtain sufficient health awareness resources, their food choices tend to favor healthy products, thereby reducing impulsive eating behavior [72–74]. In fact, children have fewer intellectual resources in many fields compared to adults [75, 76], and their understanding of product prices and the value of goods and services are not mature enough [77, 78]; therefore, with limited cognitive abilities and experience, children often lack the requisite knowledge for recognizing the health implications of food.

In addition, advertisements of unhealthy products seriously affect children’s correct healthy diet and nutritional concepts. Children are susceptible to external interference (for example, TV commercials, celebrities, cartoon characters, etc.). Food promotion directly affects children’s nutritional knowledge, preferences, purchasing behavior, consumption patterns, and diet [79]. In order to better sell snacks, many businesses use promotional activities to increase consumers’ willingness to buy. For example, TV commercials often use animation to effectively attract young children and enhance their perception of food pleasure [80]. When children see a favorite cartoon character or TV star advertising a certain snack, the children will not care whether or not the snack is healthy. Instead, these advertisements prompt them to associate snacks with pleasant things; this means that in the face of unhealthy food, consumers who prefer a certain orientation ignore the detailed nutritional information on the product [79, 81, 82]. Therefore, a healthy eating attitude is important to support the choice of healthy products. Consumers with health goals will understand the nutritional information before considering the product [83], which is the correct dietary concept.

### Children living in rural areas have weak health awareness on snack packaging

This study confirms that the health awareness of children living in cities is indeed higher than that in rural areas. Past studies have shown that urban children are healthier than rural children [30–35]. While rural areas are usually not the direct cause of health literacy gaps, this gap still exists. Many of the children who grew up in the countryside were brought up by their elders who may be particularly ignorant of health awareness, so that rural children rarely have health concepts. In addition, income may also be one of the variables that affect the way children buy food. Because low-income people usually have less nutritional knowledge than middle- and high-income people, they face more obstacles in identifying and choosing healthy foods [35]. However, many factors are still involved in the weak health awareness of rural children. The factor of family education may be one of the potential impacts on rural areas [34, 36, 37]. Due to research limitations, this study did not investigate the educational background of children’s parents or explore their health knowledge; this may be a topic in future research.

## Conclusions

Front of Package (FOP) Nutrition Labeling can identify unhealthy foods for children and increase their alertness to unhealthy foods. This study compares GDA, TLS, Apple label, and Warning label on the packaging on the front of snacks, and examines children’s health awareness. The results of the study include three conclusions: (1) rural children’s health awareness is weak on snack packaging; (2) children who often buy snacks have low health awareness; (3) GDA, TLS and Apple labels can help children determine healthier food choices.

Although this study was not conducted in a real retail environment, these snack products have always been favorites for children in Taiwan. The possible limitations of this study lie in three items: (1) This study cannot fully understand the family background, eating habits, parental rearing styles, and food types of each child. (2) Because, the factors that affect children’s unhealthy diet involve a very wide range, the use of labelled snack packaging composite images to allow children to make judgments may be subjective and may not fully represent the opinions of all children. Judging from the current results, it was possible to find the effectiveness of the designed Apple label for health reminders; and (3) This research result is limited to the snack packaging category and cannot be fully applied to other categories.

## Supplementary Information

**Additional file 1.** Sample of snack packaging (the % Daily Value (DV) in parentheses)

**Additional file 2.**

**Additional file 3.**

**Additional file 4.**

**Additional file 5.**

**Additional file 6.**

**Additional file 7.**

**Additional file 8.**

**Additional file 9.**

**Additional file 10.**

**Additional file 11.**

## Data Availability

All data generated or analysed during this study are included in this published article [and its supplementary information files].
