# Supplementary material for: Effects of food nutrition labels on the health awareness of school-age children
Source: BMC Public Health. 2022 Jun 24;22:1249. doi: 10.1186/s12889-022-13613-y (PMC9230083; doi:10.1186/s12889-022-13613-y)
Supplement: Supplementary file 1 — Additional file 1. Sample of snack packaging (the % Daily Value (DV) in parentheses) [file 12889_2022_13613_MOESM1_ESM.docx]

*Additional file 1* Sample of snack packaging (the % Daily Value (DV) in parentheses)

| No. | Product name | Example | Calories  kcal/per | Total Fat  g/per bag | Sat. Fat  g/per bag | Sugars  g/per bag | Sodium  g/per bag |
| --- | --- | --- | --- | --- | --- | --- | --- |
| 1 | 可樂果  (Kola Nut) | 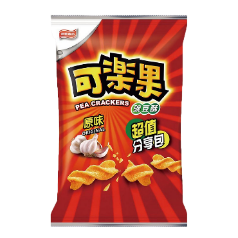 | 125  (6.3%) | 6.9  (11.5%) | 3.1  (17.2%) | 0.4  (0.8%) | 0.13  (6.7%) |
| 2 | 乖乖  (Kuai Kuai) | 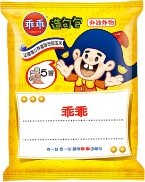 | 129  (6.5%) | 6.2  (10.3%) | 2.4  (13.3%) | 1.2  (2.4%) | 0.17  (8.7%) |
| 3 | 乖乖QQ水果軟糖  (Kuai Kuai: QQ Fruit Jelly) | 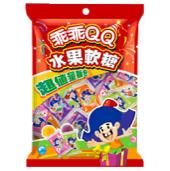 | 34  (1.7%) | 0  (0%) | 0  (0%) | 8.2  (16.4%) | 0.01  (0.3%) |
| 4 | 樂事洋芋片(原味)  (Lay's Potato Chips: Original) | 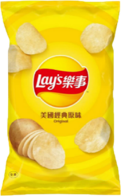 | 123  (6.2%) | 8.8  (14.7%) | 4.4  (24.4%) | 0  (0%) | 0.14  (7.1%) |
| 5 | 科學麵  (Science Noodles) | 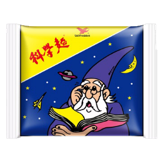 | 72  (3.6%) | 3.0  (5%) | 1.4  (7.8%) | 0.4  (0.8%) | 0.18  (9%) |
| 6 | 金莎巧克力  (Ferrero Rocher Chocolate) | 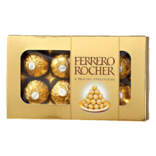 | 75  (3.8%) | 5.3  (8.9%) | 1.8  (10%) | 5  (10%) | 0.01  (0.4%) |
| 7 | 奇多(起司)  (Cheetos: Cheese) | 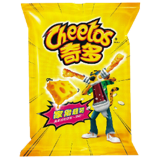 | 147  (7.4%) | 9.4  (15.7%) | 5.0  (27.8%) | 0.9  (1.8%) | 0.19  (9.3%) |
| 8 | Pocky (巧克力)  (Pocky: Chocolate) | 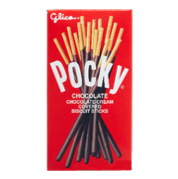 | 197  (9.9%) | 8.2  (13.7%) | 4.9  (27.3%) | 12.1  (24.2%) | 0.08  (3.8%) |

Note: The % Daily Value (DV) tells you how much a nutrient in a serving of food contributes to a daily diet. 2,000 calories a day is used for general nutrition advice.
